# Clinical Note Generation From Doctor-Patient Conversations Using Parameter-Efficient Fine-Tuning Large Language Models: Comparative Study

**DOI:** 10.2196/82545

**Published:** 2026-06-03

**Authors:** Saib Ahmed, Farig Yousuf Sadeque

**Affiliations:** 1 Department of Computer Science & Engineering, BRAC University, Kha 224 Pragati Sarani, Merul Badda, Dhaka, 1212, Bangladesh, 880 1796965173

**Keywords:** natural language processing, clinical natural language processing, clinical NLP, Dialogue2Note, transformer, decoder-only, Mistral, Llama, Meditron, summarization, Recall-Oriented Understudy for Gisting Evaluation, Recall-Oriented Understudy for Gisting Evaluation score, ROUGE score, bidirectional encoder representations from transformers, bidirectional encoder representations from transformers score, BERTScore

## Abstract

**Background:**

Clinical note documentation is a vital yet time-intensive task in health care. While advancements in natural language processing have transformed many domains, generating accurate summaries of doctor-patient conversations remains underexplored due to the limited availability of open-source datasets. Large language models (LLMs), with their training on vast datasets, present a promising solution to this challenge.

**Objective:**

Precision in clinical summarization is crucial, as it directly impacts patient care and safety. This study aimed to evaluate the effectiveness of parameter-efficient, fine-tuned, decoder-only LLMs for clinical note generation from doctor-patient conversations. We focus on assessing medical accuracy, robustness, and the feasibility of parameter-efficient fine-tuning (PEFT) approaches under practical resource constraints.

**Methods:**

We used the Medical Training Summarization Dialog dataset containing 1700 doctor-patient conversations paired with clinical notes. Several decoder-only LLMs, including Mistral, Meditron, and Llama, were fine-tuned using PEFT techniques to reduce computational and memory overhead. Evaluation was performed using standard automatic metrics, including the Recall-Oriented Understudy for Gisting Evaluation score and bidirectional encoder representations from transformers score, to assess content overlap and semantic similarity between generated and reference clinical notes. In addition, an expert physician assessed the LLM-generated notes for medical accuracy, completeness, concision, relevance, and clinical coherence and readability.

**Results:**

Model performance was evaluated using the Recall-Oriented Understudy for Gisting Evaluation score and bidirectional encoder representations from transformers scores, demonstrating that Meditron-7B and Llama3-8B achieved state-of-the-art results among open-source, parameter-efficient, fine-tuned models, with Mistral-7B also performing competitively. The findings indicate that decoder-only LLMs, particularly Llama variants, outperform traditional models. Moreover, fine-tuning with higher quantization has the potential to further enhance performance. Human expert evaluation further indicated that Llama3-8B and Mistral-7B produced clinically coherent and accurate summaries, with Meditron-7B and Llama3-3B also performing reliably across evaluation criteria. The findings suggest that higher quantization during fine-tuning may improve efficiency without substantially compromising performance.

**Conclusions:**

This study underscores the potential of the PEFT of decoder-only LLMs to transform clinical workflows by streamlining medical documentation, thereby enabling health care professionals to dedicate more time to patient care. These models offer a scalable and resource-efficient alternative to traditional architectures and have the potential to streamline clinical documentation workflows.

## Introduction

### Background

Manually creating clinical notes has always been a time-consuming and exhausting task for health care providers. As health care systems grow increasingly complex and large-scale, the need for faster and more accurate documentation methods has become more pressing. Transformer architecture has brought significant advancements to various natural language processing (NLP) tasks, including text summarization—a fundamental task in NLP. These advancements, driven by transformer-based large language models (LLMs) and the availability of large-scale datasets, have the potential to revolutionize health care systems. An NLP-powered system can analyze doctor-patient conversations, identify relevant clinical facts, structure the information, and generate coherent medical reports. By automating the generation of clinical notes, such systems provide timely insights and support to medical professionals during patient interactions. Real-time information retrieval ensures clinicians have immediate access to relevant medical data and patient histories, which can aid in making critical decisions. This, in turn, leads to more accurate diagnoses, personalized treatment strategies, and improved patient outcomes. Beyond individual patient interactions, the ability to analyze data at scale enables medical facilities to make data-driven decisions that enhance overall treatment quality, optimize resource use, and improve patient satisfaction. These innovations promise to streamline health care processes and elevate the standard of care.

The challenge lies in ensuring these automated notes are precise. Any mistakes or *hallucinations* in medical facts could have serious consequences. Summarizing clinical dialogues is tricky, but we tackled this by using decoder-only transformer models, which we found outperform traditional sequence-to-sequence models (such as Flan-T5-Large) on metrics such as Recall-Oriented Understudy for Gisting Evaluation (ROUGE) and bidirectional encoder representations from transformers (BERT) scores. Using the Medical Training Summarization Dialog (MTS-Dialog) dataset [1] containing 1700 doctor-patient conversations and their summaries, we explored models such as Mistral [2] and Llama [3]**,** with Llama3 [4] emerging as the top performer, even beating the best results from the 2023 MEDIQA-Chat challenge [5].

### Motivation

Manual note-taking can be time-consuming, diverting health care providers’ attention from patient care. On average, physicians dedicate approximately 52 to 102 minutes each day documenting clinical notes based on their patient interactions [6]. Automatic clinical note generation can be a solution to this problem. It can reduce the burden of paperwork on health care providers and improve the accuracy of the medical records. This allows doctors to focus more on patient care rather than on paperwork. During the COVID-19 pandemic, face-to-face doctor visits were restricted. For that reason, health care systems experienced over a 100% surge in virtual urgent care appointments and more than a 4000% rise in virtual nonurgent care visits [7]. Automatic clinical note generation can help us to overcome this kind of situation. Clinical notes can vary widely in terms of content, format, and quality. Automated systems can help to standardize documentation, improve data quality, and facilitate analysis. Nevertheless, automated systems can extract valuable insights from clinical notes, enabling data-driven decision-making and improving patient care.

### Research Objective

Our research set out to fine-tune the LLMs to craft high-quality clinical notes that make a real difference. We aimed to find a model that balances speed and precision, fine-tuning it on MTS-Dialog to adapt to the dataset’s unique demands. Our ambition was to go beyond the current leader, Flan-T5-Large, and establish a new benchmark for automated documentation. To measure our success, we compared our model’s ROUGE and BERT scores against Flan-T5-Large, assessing its ability to summarize accurately while preserving every essential detail.

### Literature Review

The study by Ben Abacha et al [1] introduced the MTS-Dialog dataset, pairing simulated doctor-patient dialogues with clinical notes. They tested transformer models such as Bidirectional and Auto-Regressive Transformers (BART) [8] and Pegasus [9], finding BART, especially when prefinetuned and guided, produced the most accurate notes. However, issues such as hallucinations and missing key details persisted, highlighting both the promise of automation in health care documentation and the need for better factual accuracy. The study by Ozler and Bethard [10] explored LLMs for summarizing medical dialogues in the MEDIQA-Chat 2023 competition. Using models such as Clinical-T5 and Roberta-base on Medical Information Mart for Intensive Care datasets, they hit a peak accuracy of 72.3%. Limited hardware and dataset size posed challenges, but their work shows LLMs’ potential for medical documentation, with room for improvement through advanced models and techniques. The study by Sharma et al [11] tackled the same competition, fine-tuning BART-large on datasets such as Medical Information Mart for Intensive Care-IV-Note and introducing an N-pass strategy to summarize long dialogues. Data augmentation (DA) with synthetic dialogues boosted results, though hallucinations remained an issue. Their research pushes clinical NLP forward, suggesting future integration of medical knowledge. The study by Wang et al [12] used ChatGPT and BioMedLM in a doctor-patient loop system for MEDIQA-Chat 2023, excelling in dialogue generation and note summarization. While effective, gaps in medical knowledge and handling lengthy conversations need work, pointing to future refinements in segmentation and expertise. The study by Suri et al [13] evaluated transformer models such as Bio-Bart and DialogLED for the same challenge. DialogLED-Large outperformed GPT-3, offering a cost-effective alternative despite limited training data. They recommend DA to improve reliability. The study by Tang et al [14] fine-tuned BART and CONFIT while leveraging GPT-4 for MEDIQA-Chat 2023. GPT-4’s natural outputs impressed human experts, though privacy concerns with external application programming interfaces surfaced. Their findings underscore LLMs’ role in streamlining clinical notes. The study by Srivastava [15] tested local-sparse-global BART ensemble methods, finding that section-wise models outperformed single approaches for chart note summaries. Multilayer techniques and PubMed fine-tuning fell short, suggesting specialization as a key focus moving forward. The study by Milintsevich and Agarwal [16] fine-tuned FLAN-T5 and LongT5, using multitask learning to cut hallucinations in clinical notes. While effective, adding clinical named entity recognition tags unexpectedly hurt quality, indicating a need for better augmentation strategies. The study by Zhang et al [17] combined an SVM classifier with GPT-3 for summarization, with GPT-3.5 outperforming a fine-tuned Curie model. Their hybrid approach hints at the power of blending traditional and modern techniques. Finally, the study by Mathur et al [18] used GPT-4 with in-context examples, topping MEDIQA 2023 rankings. Despite concise outputs, brevity and privacy risks remain challenges, marking a step forward in applying LLMs to health care. In another research, the study by Heilmeyer et al [19] demonstrated that hospitals can use smaller, open-source LLMs running on their own computers to handle sensitive documentation, rather than relying on commercial systems. They found that a model specifically optimized for the local language (German) performed exceptionally well, generating medical reports that doctors rated as usable 93% of the time. The study by Savage et al [20] showed that while basic fine-tuning is enough for simple medical checklist tasks, using the more advanced direct preference optimization method allows LLMs to handle complex clinical reasoning and triage by teaching them to distinguish between high-quality and poor responses.

## Methods

### Ethical Considerations

This study uses the MTS-Dialog dataset [1], a publicly available collection of simulated doctor-patient conversations. As the dataset contains no protected health information or real patient interactions, this research does not classify as human participants research. A qualified physician participated in this study to evaluate the clinical accuracy of the generated notes. This involvement was strictly in a professional capacity as a domain expert to assess text quality, rather than as a research participant. No personal data were collected from the evaluator.

### Main Dataset

#### Overview

There are around 1700 brief doctor-patient conversations paired with clinical notes and summaries in the MTS-Dialog dataset. The main aim behind creating this dataset is to help researchers create tools that automatically summarize doctor-patient conversations and generate clinical notes [1]. The training set comprised 1201 pairs for model training. The validation set comprised 100 pairs for fine-tuning. The test sets comprised 2 sets of 200 pairs each: test set 1 (used in MEDIQA-Chat 2023, task A) and test set 2 (used in MEDIQA-Sum 2023, tasks A and B). MEDIQA-Chat 2023 tasks encompassed task A: predict section headers (eg, HISTORY of PRESENT ILLNESS) and content from short dialogues, task B: generate complete clinical notes from full conversations, and task C: create synthetic dialogues from clinical notes. MEDIQA-Sum 2023 tasks encompassed task A: generate clinical note summaries from dialogues and task B: produce section-specific summaries (eg, ASSESSMENT and PLAN).

#### Section-Header Categories

The MTS-Dialog dataset is divided into 20 categories of section headers: fam/sochx, genhx, pastmedicalhx, cc, pastsurgical, allergy, ros, medications, assessment, exam, diagnosis, disposition, plan, edcourse, immunizations, imaging, gynhx, procedures, other_history, and labs. The statistics of the dataset shared in the paper [1] can be found in Table 1.

**Table 1.** Descriptive statistics of the Medical Training Summarization Dialog dataset.

	Count	Values, mean (SD)	Values, maximum	25th percentile	50th percentile	75th percentile
Dialogue						
Turns	15,969	9 (8.72)	103	4	6	12
Sentences	18,406	11 (13.03)	136	4	7	14
Words	2,41,685	142 (144.03)	1951	48	88	176
Summary						
Sentences	5870	3 (4.35)	3	1	2	4
Words	81,299	48 (72.02)	48	6	18	55

#### Data Quality

The MTS-DIALOG dataset undergoes a thorough 3-step process to ensure its quality. First, only those with medical backgrounds, such as former medical scribes, were selected to serve as annotators. Second, during the early stages of their work, each annotator received one-on-one feedback from an experienced trainer to help refine their skills. Finally, after the dataset was completed, an independent validation process took place. This separate review used a grading rubric to assess how well the annotated conversations followed the guidelines and how relevant the content was to the original clinical notes. Minor corrections, such as fixing typos or filling in missing information, were made during this stage to make sure the final dataset was even more accurate than the initial version [5].

#### Comparison With Real Data

The MTS-Dialog dataset includes both real medical notes and simulated conversations that mirror doctor-patient interactions, helping to avoid any breaches of confidentiality. To understand the impact of relying heavily on synthetic data, a blind review was conducted to compare the MTS-Dialog data with real conversations. Distinguishing between the simulated and real data in the dataset is a challenging task. While statistical analysis shows that the simulated conversations have fewer speech errors and pauses, medical experts noted that the dialogues generally feel authentic. In some cases, the clarity, directness, and ease of understanding, even with sudden shifts in topics, made synthetic data mistaken for real interactions. On the other hand, actual data, known for their honesty and minimal speech flaws, were often confused for simulated content due to its polished nature. This difficulty highlights the dataset’s value as a foundation for training and evaluating models in practical, real-world settings.

Back-translation augmentation stands out as a valuable method. It involves converting the original text into another language and then translating it back into the original language. This process introduces natural linguistic variations while preserving the core meaning, thus expanding the training dataset and helping models generalize better to unseen data. To reduce translation errors, French and Spanish were chosen because their vocabulary is similar to English, and they have high-performing translation models [21]. It was implemented using the following three steps: (1) translation: the original text is translated from its source language (English) to a target language (French and Spanish) using a machine translation model, (2) back-translation: the translated text is then translated back to the original language (English) using another machine translation model, and (3) augmentation: the back-translated text is added to the original training dataset, creating a larger and more diverse corpus.

After DA, the dataset includes 3.6k pairs of medical dialogues and their corresponding summaries, generated from an initial 1200 training pairs through back-translation using French and Spanish, as outlined in the study [1]. Back-translation can significantly increase the size of a training dataset. Theoretically, it can improve the performance of the summarization model. By exposing models to different linguistic variations, back-translation can help them generalize unseen data better. Nevertheless, by increasing the diversity of training data, back-translation helps prevent overfitting, which is a common problem in NLP.

### Fine-Tuning Techniques

In most cases, the graphics processing unit (GPU) hardly has enough memory to fine-tune any decoder-only LLM. To overcome this problem, we used the parameter-efficient fine-tuning (PEFT) technique proposed in the study by Houlsby et al [22]. The authors in the paper proposed a parameter-efficient transfer learning technique for NLP that introduces small trainable adapter modules into every layer of a pretrained model with frozen original model weights. The technique minimizes trainable parameters, leading to efficient and scalable fine-tuning across various tasks. We also used 8-bit quantization, where the original pretrained weights of the model are quantized to 8-bit and kept fixed during fine-tuning. This method is known as quantized low-rank adapter (QLoRA) [23].

### Research Methodology

#### Overview

**Figure 1.**
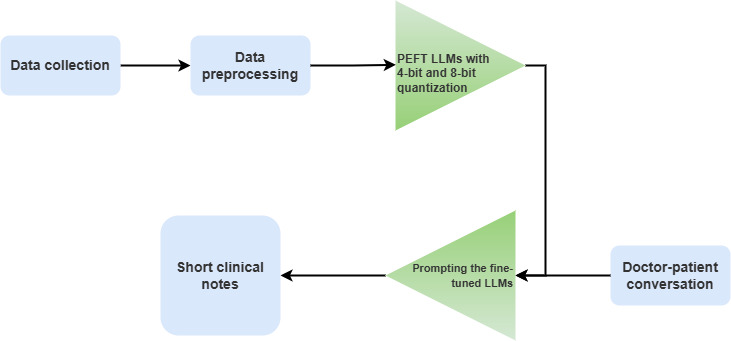
Training and inference diagram of the clinical note generation model. LLM: large language model; PEFT: parameter-efficient fine-tuning.

This research introduces a new task in text generation. To solve this, a text-generation model must be created to generate clinical notes from the conversation between doctor and patient Textbox 1. A training-inference diagram of this text-generation model is shown in Figure 1.

Textbox 1. **Algorithm 1: training procedure for clinical note generation.**

**Given dataset:** *C,N, where C={C_0_,C_1_,...,C_i_} [set of doctor-patient conversations] and N={N_0_,N_1_,...,N_i_} [set of clinical notes]*

**Objective:** learn a generative model *F* (*C_j_*) such that it produces a valid *N_j_*, where *N_j_* ∉ {*N_0_*,*N_1_*,...,*N_i_*} and *N_j_* is not generated from {*C_0_*,*C_1_*,...,*C_i_*}

A valid generated note *N_k_* must follow the syntax of the target language, maintain clinical integrity, follow the semantics of the target language, and not be a hallucination.

#### Data Preprocessing

The dataset was passed through the following preprocessing steps:

- Removing unnecessary spaces: some unnecessary tags in the dataset were removed by an empty string. Certain spaces and line gaps were eliminated for text processing and analysis to streamline the training.
- Tokenization: tokenization is one of the most vital steps in this research. In this particular research, HuggingFace models were mostly used. For this reason, HuggingFace’s AutoTokenizer class is used for tokenization.

#### Evaluation Metrics

To ensure a robust assessment of the generated clinical notes, we used both automated quantitative metrics and qualitative human expert evaluation:

- ROUGE: this metric measures the lexical overlap between the machine-generated notes and the reference summaries. We report ROUGE-1 (unigram overlap), ROUGE-2 (bigram overlap), and ROUGE-L (longest common subsequence) [24].
- BERTScore: This metric evaluates semantic similarity by leveraging contextual embeddings to compare the generated text with the reference text, capturing meaning beyond exact word matches [25].
- To validate clinical utility, a qualified physician evaluated a subset of the generated notes. The evaluation followed a 5-point Likert scale (where 1 is the lowest and 5 is the highest) across five critical clinical dimensions: (1) medical accuracy: verification of the correctness of clinical facts, (2) completeness: ensuring no vital information from the dialogue was omitted, (3) conciseness and relevance: assessing the model’s ability to filter out nonessential information, (4) clinical coherence and readability: evaluating the logical flow and professional tone of the note, and (5) overall clinical quality: an aggregate score representing the general utility of the note in a professional setting.

#### Training Setup

We used the online platform Kaggle to fine-tune our dataset. NVIDIA Tesla T4 GPU was used for both the computationally intensive fine-tuning process and the inference phase. The hardware configuration was as follows: Tesla T4, CUDA version: 12.4, and available RAM: 29 GB.

#### Fine-Tuning the Llama and Mistral Variants

At first, we tried different sequence-to-sequence models with DA techniques to beat the current state-of-the-art (SOTA) model, but the result was not satisfactory. Next, several SOTA decoder-only models, such as variants of Mistral and Llama, were evaluated. The “Meta-Llama-3-8B” model, an updated version of the Llama family with 8B parameters, outperformed the SOTA Flan-T5-Large model in the ROUGE and BERT metrics, while the “Mistral-7B-v0.3” outperformed in the BERT metric only. A smaller variant of the Llama family “Llama-3.2-3B” was also fine-tuned. Due to low hardware resources, full model fine-tuning was not feasible. We have also used “Meditron-7B.” It is a suite of open-source medical language models with 7B and 70B parameters, addressing gaps in medical artificial intelligence [26]. Built on Llama-2, the models are pretrained on a curated medical corpus, including PubMed articles and guidelines, using Nvidia’s Megatron-LM framework. We used the 7B variant of the model in this research. The decoder-only models were fine-tuned using PEFT [22] with the low-rank adaptation (LoRA) [27] method. Eight-bit quantization was used while loading the models. This method is known as QLORA [23]. In this approach, the model’s original pretrained weights are converted to 8-bit formats and remain unchanged during fine-tuning. A small number of trainable parameters, called low-rank adapters, are added during the fine-tuning process [27]. These adapters are trained to adjust the pretrained model for the specific task during fine-tuning using the 32-bit floating-point format. During computations, such as forward and backward passes in training or during inference, the 8-bit quantized weights are converted back to 32-bit floating-point numbers. After fine-tuning, the model includes the original weights in their 8-bit form, along with the low-rank adapters in their higher-precision format. A significant percentage of this research and analysis is devoted to fine-tuning. For this procedure, the MTS-Dialog dataset was used. Meta-Llama-3-8B, Llama-3.2-3B, Meditron-7B, and Mistral-7B-v0.3 LLMs were used for fine-tuning with the following hyperparameters. All the models were loaded with 8-bit quantization, and the following LoRA hyperparameter configurations were maintained and described in Table 2. The prompt that was used as a prefix to summarize the doctor-patient dialogue to generate clinical notes was as follows:

Summarize the following patient-doctor dialogue. Include all medically relevant information, including family history, diagnosis, past medical (and surgical) history, immunizations, lab results, and known allergies.

This primary prompt was used to generate all quantitative performance metrics reported in Table 3. For the evaluation of our fine-tuned models, we calculated performance metrics independently for test set 1 and test set 2. These independent scores were subsequently averaged to ensure comparability with baseline models reported in the literature. Furthermore, we explicitly confirm that there is no data overlap between the training set and these test sets.

**Table 2.** Hyperparameter settings used for fine-tuning the large language models, including parameters for training arguments and generation. Detailed configuration of the training environment and model parameters used to adapt Mistral, Llama, and Meditron variants for clinical note generation.

Hyperparameters	Settings
per_device_train_batch_size	1
logging_steps	100
warmup_steps	0.03
save_strategy	epoch
group_by_length	True
lr_scheduler_type	constant
max_seq_length	512
lora_alpha	16
lora_dropout	0.1
LoRA attention dimension (rank), r	64
target_modules	q_proj, k_proj, v_proj, o_proj, gate_proj, up_proj, down_proj, lm_head
bias	none
task_type CAUSAL_LM	CAUSAL_LM
max_new_tokens	512
do_sample	True
temperature	0.8
pad_token_id	tokenizer.eos_token_id

**Table 3.** Performance metrics for fine-tuned large language models on the clinical note generation task, measured by Recall-Oriented Understudy for Gisting Evaluation (ROUGE) and bidirectional encoder representations from transformers score (BERTScore).

Method	ROUGE-1	ROUGE-2	ROUGE-L	BERTScore-F1
Llama-3.2-3B	0.3686	0.1517	0.2895	0.8901
Llama-3-8B	*0.4574^a^*	*0.2079*	*0.3636*	*0.9060*
Llama-3-8B (DA^b^)	0.4131	0.1888	0.3410	0.8956
Mistral-7B	0.3889	0.1431	0.3184	0.8933
Mistral-7B (DA)	0.3679	0.1112	0.3014	0.8898
Meditron-7B	*0.4560*	0.1931	*0.3667*	*0.9056*

a Best scores are italicized.

b DA: data augmentation.

While training, the following “TrainingArguments” class’s hyperparameter configuration was used, shown in Table 2. Given the severe computational and memory constraints of fine-tuning LLMs on a single Tesla T4 GPU, conducting an exhaustive algorithmic hyperparameter search (such as a grid search) was computationally prohibitive. Instead, we used an empirical, manual tuning strategy. We iteratively tested a targeted subset of hyperparameter combinations based on established best practices for PEFT. For example, we evaluated LoRA α values of 8, 16, and 32, observing empirically that an alpha of 16 provided the most stable validation loss and convergence speed for our dataset without triggering out-of-memory errors. Pytorch “generate()” method generates clinical notes from the fine-tuned LLMs. The hyperparameters used for generating clinical notes are also shown in Table 2. These same hyperparameters were maintained for all the models that are used in this research.

We used a maximum sequence length of 512 tokens due to hardware memory constraints. Though the dataset contains dialogues exceeding this length, truncation was applied to fit the inputs within this limit.

## Results

The results are shown after fine-tuning the Llama3-8B and Mistral-7B models in Table 3. ROUGE [24] and BERTScore [25] were used as evaluation metrics. The performances with and without DA are displayed in the table. The fine-tuned Llama-3-8B and Meditron-7B models achieved comparable SOTA performance. Llama-3-8B attained a ROUGE-1 score of 0.4574 and BERTScore-F1 of 0.9060. Notably, Meditron-7B achieved a slightly higher ROUGE-L score (0.3667 vs 0.3636), suggesting strong performance in structural coherence, likely due to its domain-specific pretraining. Given the marginal differences in ROUGE-1 scores (0.0014 difference), we classify both models as top-tier performers among the open-source variants tested, rather than declaring a single definitive winner.

For some reason, Llama-3-8B with DA performs lower than this. With DA, we get a ROUGE-1 score of 0.4131, a ROUGE-2 score of 0.1888, a ROUGE-L score of 0.3410, and a BERTScore-F1 score of 0.8956. A smaller version of the Llama model, “Llama-3-3B,” is also fine-tuned, but the performance was not good enough, indicating that model parameter size remains a critical factor for this specific task. The model gave ROUGE-1 score of 0.3686 and BERTScore-F1 of 0.8901. The fine-tuned Mistral model also delivered competitive results, achieving a BERTScore-F1 of 0.8933. However, in the case of ROUGE scores, it lags behind Llama3. With DA, the performance of the Mistral-7B model decreased.

An expert physician evaluated the LLM-generated clinical notes using a 5-point Likert scale (1-5) across five dimensions: medical accuracy, completeness, conciseness and relevance, clinical coherence and readability, and overall clinical quality (average of all these criteria). Higher scores indicate better performance across all criteria. The human evaluation result is shown in the Table 4.

**Table 4.** Physician-rated clinical quality evaluation of the large language model–generated notes. A qualitative assessment conducted by a qualified domain expert to validate the clinical utility of the models. Five key dimensions were evaluated using a 5-point Likert scale: medical accuracy, completeness, conciseness and relevance, clinical coherence and readability, and overall clinical quality.

Model	Medical accuracy, mean (SD)	Completeness, mean (SD)	Conciseness and relevance, mean (SD)	Clinical coherence and readability, mean (SD)	Overall clinical quality, mean (SD)
Llama-3.2-3B	4.29 (1.14)	4.38 (1.02)	4.43 (1.05)	4.65 (0.91)	4.44 (0.93)
Llama-3-8B	4.66 (0.86)	4.65 (0.89)	4.73 (0.82)	4.83 (0.74)	4.72 (0.75)
Mistral-7B	4.55 (0.68)	4.68 (0.60)	4.73 (0.60)	4.89 (0.42)	4.71 (0.51)
Meditron-7B	4.43 (0.90)	4.47 (0.90)	4.73 (0.55)	4.89 (0.35)	4.63 (0.52)

## Discussion

### Principal Findings

The results of this study demonstrate that decoder-only transformer models, specifically Llama3-8B and Meditron-7B, achieve SOTA performance among open-source, parameter-efficient, fine-tuned models in generating clinical notes from doctor-patient dialogues. Our findings indicate that PEFT with 8-bit quantization (QLoRA) allows these large models to perform effectively under hardware constraints while maintaining high medical accuracy. The results clearly show that DA did not enhance the performance of either model; both the Mistral and Llama variants performed better without it. A similar phenomenon has been noted and explained in a previous research paper [28]. Notably, an expert physician’s evaluation confirmed that these models produce clinically coherent and accurate summaries, with Llama3-8B and Mistral-7B leading in overall clinical quality.

### Comparison With Prior Work

Our research establishes a new benchmark by surpassing the previous leader, Flan-T5-Large, in automatic evaluation metrics such as ROUGE and BERTScore. Unlike some entries in the MEDIQA-Chat 2023 competition that relied on DA to boost performance, our experiments showed that DA actually decreased performance for Mistral and Llama variants, aligning with findings in other specialized medical NLP research.

### Limitations

Table 5 shows the training and inference times for different models. Training time is measured in seconds/epoch. All fine-tuned models are inferred with a Tesla T4 GPU. From Table 5 it is visible that the inference time for Mistral-7B is better than Llama-3-8B, but it is worse than Meditron-7B. However, for real-world applications, it needs to be improved further. Training time for both models is quite long.

**Table 5.** Computational efficiency and latency analysis per clinical note generation. This table compares the training duration (seconds/epoch) and average inference latency (seconds/note) across the four primary model architectures. Conducted on a Tesla T4 graphics processing unit, these metrics evaluate the feasibility of deploying decoder-only large language models in real-time clinical settings where documentation speed is critical for reducing physician burnout.

Model	Training time	Average inference time
Llama-3.2-3B	1406.6	12.49
Llama-3-8B	2588.9	13.41
Mistral-7B	2171.6	8.68
Meditron-7B	2566.06	6.27

All the fine-tuned models had some gender biases. As the “MTS-Dialog” dataset is a short doctor-patient conversation dataset, sometimes it is quite difficult to understand the patient’s gender from the conversation. In these situations, all of the fine-tuned models mostly assume the patient’s gender as male. In a few cases, the model predicts the gender as female, while in the annotation, the patient is identified as male. In some cases in the dataset, the gender pronoun is used incorrectly. We assume that most of the model’s pretraining dataset could be biased, which is causing this type of problem. In some cases in the dataset, the gender pronoun is used incorrectly. To overcome this, we use the following new prompt during inference:

Summarize the following patient-doctor dialogue. To ensure a comprehensive summary, follow these steps:

Gender identification: identify the patient’s gender based on the context and use appropriate pronouns throughout the summary.

Medical history: summarize the patient’s family history, past medical and surgical history, and known allergies. Ensure each detail is categorized.

Current visit: identify the main concerns discussed, including symptoms, lab results, and diagnosis.

Immunizations: list any relevant immunization history.

Plan and recommendations: note any treatment plan, medications prescribed, or follow-up actions recommended by the doctor. Ensure your summary flows logically, preserving the order of the conversation, while focusing on medically relevant details.

However, even after using this prompt, there was still no significant improvement. This gender-aware prompt was evaluated qualitatively but is not reflected in these baseline quantitative results.

Another major limitation of our quantitative evaluation is the reliance on a single expert physician, which introduces potential evaluator bias and precludes the calculation of interrater reliability metrics such as Cohen κ. Future studies should use multiple independent evaluators to mitigate individual bias.

### Future Work

The amount of data in the medical domain are very limited. It is also very hard to get access to this type of data because of the physician-patient privilege. Most of the patients are not comfortable sharing their private data. It is essential to develop more data, which will make it easier to create an automatic clinical note generation system. The dataset used in this research is short conversations between doctors and patients. A more and longer real-world doctor-patient conversation corpus is needed in the future to improve the quality of clinical note generation.

Domain-specific pretrained decoder-only LLMs have improved the domain-specific task a lot in recent years. A pretrained model such as Code Llama is one example in the coding domain [29]. Developing a decoder-only model that is pretrained on a large medical corpus might help to create clinical note generation tasks.

In diverse health care settings, doctors and patients may converse in different languages. A cross-lingual summary can help bridge this language gap by automatically summarizing conversations in one language and translating the summary into another, making it accessible to a wider range of health care providers. Creating a cross-lingual clinical note generator could greatly impact this domain.

In this research, text data from doctor-patient conversations are used. Generating clinical notes directly from the spoken interactions between doctors and patients could provide a more accurate and practical solution.

The maximum sequence length was set to 512 tokens due to hardware memory constraints. This truncation represents a methodological limitation that may have affected the completeness of the generated notes for longer dialogues. Future implementations should consider sliding window approaches to handle lengthy conversations without information loss.

It is also shown that just updating the prompt while doing inference is not a solution to this problem. The hypothesis of the authors of this paper suggests these steps to overcome the problem. The wrongly addressed gender in the dataset should be corrected manually in the reference note, and the patient’s gender information should be included in the conversation. After that, the prompt should be updated by including the information to detect the correct gender of the patient and use this prompt to fine-tune the model. By doing all these tasks, it may be possible to solve the problem.

### Conclusions

In this research, we experimented with various decoder-only transformer architectures to fine-tune models for generating clinical notes by summarizing conversations between doctors and patients. The results demonstrate that decoder-only models such as Llama3 and Mistral outperform classical encoder-decoder models such as Flan-T5 and Pegasus in summarizing medical discussions. Larger models tend to deliver better results compared to smaller ones. By fine-tuning the Llama-3-8B and Meditron-7B models, SOTA performance among open-source, parameter-efficient models was achieved in terms of ROUGE-1 score and BERTScore. However, none of the models underwent full fine-tuning. Instead, PEFT methods were applied across the board. The study highlights the importance of domain-specific pretraining and high-quality annotated datasets. While our methods show promising results, there are still challenges to overcome, such as hardware limitations, gender bias, and the lack of diverse medical datasets. Ultimately, automating clinical documentation through decoder-only LLMs has the potential to enhance health care efficiency. However, given the unresolved gender bias observed in this study, these models are currently best suited as assistive tools in human-in-the-loop workflows rather than autonomous systems, requiring further research into bias mitigation before they can ensure fully accurate patient records.

## Abbreviations

BART: Bidirectional and Auto-Regressive Transformers
BERT: bidirectional encoder representations from transformers
DA: data augmentation
GPU: graphics processing unit
LLM: large language model
LoRA: low-rank adaptation
MTS-Dialog: Medical Training Summarization Dialog
NLP: natural language processing
PEFT: parameter-efficient fine-tuning
QLoRA: quantized low-rank adapter
ROUGE: Recall-Oriented Understudy for Gisting Evaluation
SOTA: state-of-the-art
